# The Nanomechanical Properties of *Lactococcus lactis* Pili Are Conditioned by the Polymerized Backbone Pilin

**DOI:** 10.1371/journal.pone.0152053

**Published:** 2016-03-24

**Authors:** Mickaël Castelain, Marie-Pierre Duviau, Alexis Canette, Philippe Schmitz, Pascal Loubière, Muriel Cocaign-Bousquet, Jean-Christophe Piard, Muriel Mercier-Bonin

**Affiliations:** 1 Université de Toulouse, INSA, UPS, INP, LISBP, 135 Avenue de Rangueil, F-31077, Toulouse, France; 2 INRA, UMR792 Ingénierie des Systèmes Biologiques et des Procédés, F-31400, Toulouse, France; 3 CNRS, UMR5504, F-31400, Toulouse, France; 4 Micalis Institute, INRA, AgroParisTech, Université Paris-Saclay, 78350, Jouy-en-Josas, France; LAAS-CNRS, FRANCE

## Abstract

Pili produced by *Lactococcus lactis* subsp. *lactis* are putative linear structures consisting of repetitive subunits of the major pilin PilB that forms the backbone, pilin PilA situated at the distal end of the pilus, and an anchoring pilin PilC that tethers the pilus to the peptidoglycan. We determined the nanomechanical properties of pili using optical-tweezers force spectroscopy. Single pili were exposed to optical forces that yielded force-versus-extension spectra fitted using the Worm-Like Chain model. Native pili subjected to a force of 0–200 pN exhibit an inextensible, but highly flexible ultrastructure, reflected by their short persistence length. We tested a panel of derived strains to understand the functional role of the different pilins. First, we found that both the major pilin PilB and sortase C organize the backbone into a full-length organelle and dictate the nanomechanical properties of the pili. Second, we found that both PilA tip pilin and PilC anchoring pilin were not essential for the nanomechanical properties of pili. However, PilC maintains the pilus on the bacterial surface and may play a crucial role in the adhesion- and biofilm-forming properties of *L*. *lactis*.

## Introduction

Many bacteria, especially pathogens, produce long polymeric cell-surface organelles, called pili, that initiate bacterial attachment to host tissues, facilitating colonization and invasion [1–3]. The biogenesis, structure, and properties of these extended hair-like structures are well characterized in Gram-negative bacteria, especially for type 1, IV and P pili [1–5]. In contrast, pilus-like structures on the surface of Gram-positive bacteria were first detected in the 1960’s in *Corynebacterium renale* by electron microscopy [6] but have only recently been characterized at the molecular level [7]. A number of studies has reported the occurrence of filamentous structures in Gram-positive bacteria (see, for example, ref. [8] for review) and demonstrated significant morphological and structural differences between the pili of Gram-positive and Gram-negative bacteria [9,10]. A generic model for the assembly of pili in Gram-positive bacteria [7,11] has proposed that they are assembled from hundreds of copies of a single major pilin that forms the shaft along with one or two ancillary pilins *i*.*e*. an adhesive pilin located at the pilus tip and an anchoring pilin at the base of the pilus. Pilin subunits are secreted extracellularly and are assembled linearly by a pilus-specific class C-sortase, a transpeptidase that links the C-terminus of one subunit to the amino side chain of a lysine residue from the next pilin subunit through a covalent isopeptide bond [7,12]. Once a pilus is assembled, another sortase (usually a housekeeping sortase) ligates the anchoring pilin to an amino group of the cell wall peptidoglycan [13], highlighting the fact that these pili are entirely covalent assemblies. This architecture, typical of Gram-positive sortase-assembled pili, has been reported for several pathogens, such as *Streptococcus agalactiae* [14], *Streptococcus pyogenes* [15] or *Streptococcus pneumoniae* [16]. In contrast, data available for pili of non-pathogenic Gram-positive bacteria, such as Lactic Acid Bacteria (LAB), are scarce and restricted to *Lactobacillus rhamnosus* GG pili consisting of 1-μm long linear ultrastructures, resulting from the assembly of several copies of major pilin SpaA along with ancillary pilins [17]. The distribution of the SpaC ancillary pilin along the shaft confers the ability of the *L*. *rhamnosus* GG pilus to adhere via several attachment sites [18,19].

*Lactococcus lactis* is considered to be a major LAB present in numerous ecological niches involved in the global food chain including soil [20,21], plants [22–25], silages [26–28], milk [29,30], and fermented food products [31,32]. Some strains of *L*. *lactis* have pili [33,34]. A pilus biogenesis chromosomal cluster has been identified in in *L*. *lactis* IL1403 [34]. This *pil* operon consists of three pilin encoding genes and one sortase C gene. Over-expression of the *pil* operon results in the production and display of pili consisting of the 3 pilins *i*.*e*. the backbone-forming major pilin PilB, the pilin PilA situated at the distal end of the pilus, and the anchoring pilin PilC involved in the tethering of the pilus to the peptidoglycan [34]. These pilins are polymerized head-to-tail through isopeptide bond formation catalyzed by class C sortase for assembling pilin and the nascent pilus is anchored to the cell wall by the housekeeping sortase A [35]. Whereas the pilus biogenesis machinery in *L*. *lactis* is now well characterized [34], a comprehensive understanding of their nanomechanical properties is still lacking.

We have aimed to fill this gap using optical-tweezers force spectroscopy to unravel the respective roles of PilA, PilB and PilC pilins. We used a collection of strains expressing the native pilus operon or derivative pilus operons in which pilin and/or sortase C structural genes have been deleted or modified.

## Materials and Methods

### Ethics Statement

All experimental procedures were approved by the “Laboratoire d'Ingénierie des Systèmes Biologiques et Procédés” (Toulouse, France).

### Bacterial strains and growth conditions

**Fig 1:**
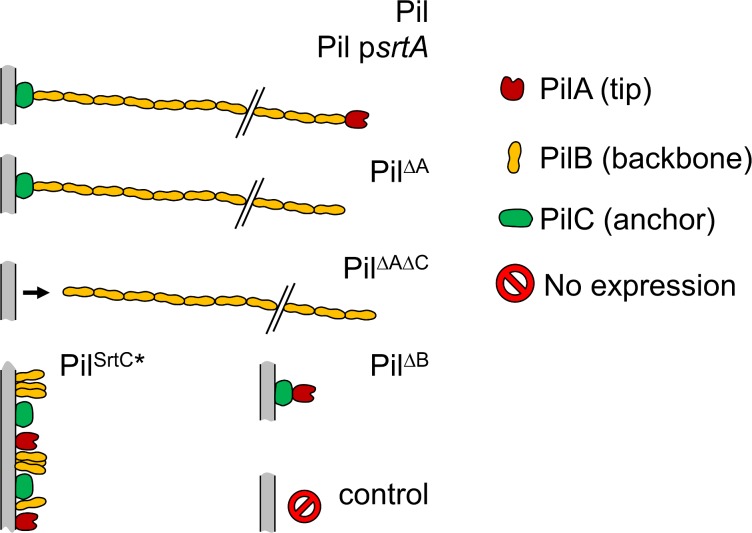
Predicted pilus topology of the strains used in this study. Scheme not to scale.

The lactococcal strains used are listed in Table 1. All are derived from *L*. *lactis* subsp. *lactis* IL1403 and have been previously described [34,36]. The predicted architecture and topology of the different gene products based on this previous work are schematically shown in Fig 1. The relative straightness of the pili has been depicted strictly for representative purposes. Briefly, the *L*. *lactis* Pil strain displays functional pili. The Pil p*srtA* strain, lacking the chromosomal sortase A gene, was complemented with the p*srtA* plasmid yielding high expression of the *srtA* gene resulting in the display of higher amounts of pili on the cell surface of this *L*. *lactis* strain than on the lactis Pil strain [36]. The Pil^ΔA^ strain was deleted for the gene for the PilA tip pilin and the Pil^ΔAΔC^ strain for the genes for both the PilA tip pilin and the PilC anchoring pilin. These four strains all produce the backbone PilB polymerized structure [34]. Additionally, the Pil^ΔB^ strain is deleted for the gene for the PilB backbone pilin and cannot produce a polymerized structure. The Pil^strC*^ strain that produces an inactive sortase C is unable to polymerize pilins.

**Table 1:** Bacterial strains and plasmids used in this study.

Strain number and strain designation hereafter	Relevant characteristic(s)^a^	Reference
*L*. *lactis* VE17061, control	Wild-type strain (WT)	[34]
*L*. *lactis* VE17173, Pil	WT, *pilA*:*pilB*:*srtC*:*pilC*	ibid
*L*. *lactis* VE17148, Pil^ΔA^	WT, *pilB*:*srtC*:*pilC*	ibid
*L*. *lactis* VE17183, Pil^ΔAΔC^	WT, *pilB*:*srtC*	ibid
*L*. *lactis* VE17190, Pil^ΔB^	Δ*pilB*, *pilA*:*srtC*:*pilC*	ibid
*L*. *lactis* VE17191, Pil^SrtC*^	Δ*srtC*, *pilA*:*pilB*:*pilC*	ibid
*L*. *lactis* VE17176, Δ*srtA* p*srtA* Pil	Δ*srtA*, p*srtA*, *pilA*:*pilB*:*srtC*:*pilC*, p*strA* (pIL2608:*strA*)	[34,36]

^a^ The host strain as well as the over-expressed *pil* operon derivatives are indicated; the *pil* operon and the topology of the predicted gene products are shown in Fig 1. Genes *yhgD*, *yhgE*, *yhhA*, and *yhhB* whose function has been characterized [34] are hereafter termed *pilA*, *pilB*, *srtC*, and *pilC*, respectively while the *ylcC* gene [36] is termed *srtA*.

SrtC* designates sortase C whose active site has been inactivated.

Bacterial stock cultures were grown in M17 broth (Oxoid) containing 0.5% (w/v) D-glucose, 5 μg.mL^-1^ erythromycin (Ery) and stored at -80°C in 20% (v/v) glycerol. Aliquots of stock cultures were first streaked on agar plates and incubated 24 h at 30°C. For the Pil p*srtA* strain, the same medium with added tetracycline (Tet) at 5 μg.mL^-1^ was used.

The bacteria were then harvested as follows: 1–2 colonies were picked from the agar plates and dispersed for approximately 5 min in phosphate-buffer saline (PBS) solution, complemented in some cases with bovine serum albumin (BSA, Sigma-Aldrich, France). 1X buffer was prepared using NaCl (140 mM), KCl (2.7 mM), Na_2_HPO_4_ (10.1 mM), and KH_2_PO_4_ (1.8 mM) pH 7.4 in Milli-Q grade water (Millipore, Billerica, USA) and filtered using a 0.2 μm-pore size filter. To determine the influence of BSA on pili interactions, 10X stock solutions with 2%, 1% and 0.1% (w/v) BSA were prepared in PBS. These solutions were dispensed into 1-mL aliquots and stored at -20°C until use. A panel of 1X BSA solutions at 0.2% (w/v), 0.1% (w/v) and 0.01% (w/v) corresponding to 30 μM, 15 μM, and 1.5 μM of BSA, respectively, were used.

To isolate the lactoccocal pili, colonies of the strain Pil^ΔAΔC^ were picked from agar plates. They were resuspended into 10 mL cold 5 mM Tris-HCl (pH 8.0). The suspension was then vortexed 3 times and centrifuged (9300 g, 30 min, 4°C). Supernatants were treated with ammonium sulfate (55% (w/v)) overnight [37,38]. The precipitated pili were harvested by centrifugation (9300 g, 30 min, 21°C), extensively dialyzed against PBS, and filtered using a 0.2 μm low-protein binding filter.

### Transmission electron microscopy of *L*. *lactis*

The ultrastructure of lactococcal pili was analyzed using transmission electron microscopy (TEM) and a negative staining procedure. All solutions were prepared extemporaneously and ultrafiltered using a 0.2 μm pore-size filter. 24-h to 48-h old colonies were picked from agar plates and resuspended in droplets of 50 μL PBS buffer. Formvar carbon coated copper grids (300 Mesh, Electron Microscopy Sciences, Hatfield, USA) were deposited over bacterial suspension droplets and left for 3 min to allow the adsorption of bacteria. Staining was performed by immersion of the grids in a 1% (w/v) pH 7 phosphotungstic acid (Sigma-Aldrich, France) solution for 20 s and the grids were dried on Whatman grade n°1 cellulose filter paper. Samples were observed at 75 kV with an H-600 TEM (Hitachi) equipped with a 1024 x 1024 pixel format Orca CCD camera (Hamamatsu, Massy, France) driven by AMT image capture engine software (version 5.42). Images were then post-processed using MATLAB^®^ software (Mathworks, Austin, USA).

### Optical tweezers set-up

Ashkin and co-corkers [39] pioneered the use of light as micromanipulators to handle micro-sized particles. Pico-Newton (pN) gradient and scattering forces are possible using a Gaussian TEM_00_ intensity mode tightly focalized by a high numerical aperture microscope objective. In this study, initially introduced by Fällman and co-workers [40], the custom-made force-measuring optical tweezers (FMOT) set-up used the main components of the Thorlabs Optical Tweezers Kit [41] (Thorlabs Inc., Newton, USA). A solid-state Nd-YAG laser source operating at 1064 nm (500 mW ultrastable, CrystaLaser, Reno, USA) was expanded using a two-lens Galilean telescope (*f* = -25 mm and *f* = 100 mm) and coupled to an oil immersion objective (Plan Fluorite, Nikon, Japan) with high numerical aperture (NA = 1.3). The pointing and intensity stability was measured over 24 hours and fitted to the manufacturer’s specifications (< 0.25% rms). To control the intensity, a half-wave (λ/2) plate mounted on a rotational motor was combined with a polarizing beam splitter cube such that the intensity of the transmitted-polarized light could be remotely modulated. The reflected light was measured using a power meter (PMD100A, Thorlabs). A 10X condenser (Plan E, Nikon, Japan) collected the laser light and its back focal plane was imaged onto a quadrant photo-diode (PDQ80A, Thorlabs) for 3D-detection of micro-sized particles [42]. In order to reach the optimum filling ratio with respect to microsphere diameter [43] and subsequent lateral and axial stiffness values, the size of the beam was modulated by translating the two-lens expander along the optical axis. This method made use of the divergence of the beam and does not require extra lenses, which would introduce additional spherical aberrations and a decrease in available laser power. The distance between the source output and the first surface of the expander was in the range 310–380 mm in our system. The beam diameter before the objective was determined using the knife-edge method in both *x*- and *y*-directions [44].

The sample, illuminated by a light-emitting diode (1W), was translated by a 3D-nanopositioning piezo-stage (NanoMax 341\M, Thorlabs) and visualized using either a CCD (DUC224C, Thorlabs) or sCMOS (Edge, PCO, Kelheim, Germany) camera. Closed-loop feedback position using a gauge reader (TSG001, Thorlabs) can achieve positional resolution of 5 nm. For large displacements (up to 4 mm), the stage was equipped with 3 stepper motors that were controlled using a USB gaming joystick and custom-LabVIEW^®^ user interface (National Instruments, Austin, USA). All optics were purchased from Thorlabs Inc. and optimized for 1064 nm and anti-reflection coated. The set-up was tested for stability in the time domain using Allan variance [45] and possible sources of noise were diminished [46].

### Single-Molecule Force Spectroscopy assays

Large 10.5-μm polystyrene beads (Polysciences, Inc. Warrington, USA)–referred to as *mounting beads* throughout the paper–were washed twice, suspended in Milli-Q grade water, sonicated and immobilized on a glass coverslip at 60°C for one hour, as described in ref. [40]. The beads were functionalized by depositing a 100-μg.mL^-1^ poly-L-lysine (Sigma-Aldrich, France) solution to provide strong electrostatic attachment with the negatively charged bacteria (electrophoretic mobility determined in ref. [47]), and incubated at 37°C. The glass slides were then dried under a laminar flow hood. Small, 1-μm latex amine-modified beads (Molecular Probes, Invitrogen)–referred to as *probe beads* throughout the paper—were washed twice and suspended in PBS (complemented with BSA in some cases). The small beads and bacterial/isolated-pili suspensions were mixed 1:1 (v/v) prior to the experiments.

A flow cell was built using two strips of Parafilm^®^, sandwiched between the bottom coverslip treated with immobilized beads and a small untreated top coverslip. This was then warmed to ~80°C for a few seconds, thereby forming a channel with a depth of approximately 150-μm. The bacterial/isolated pili and probe bead suspension was introduced into the flow cell by pipetting in one end and aspiration, using a filter paper, from the other. To avoid evaporation and convection, the two ends were sealed with vacuum grease (Dow Corning, Auburn, MI). At low trap power (30 mW before the objective), bacteria were brought into close contact with the positively charged mounting beads approximately 4.5 μm from the bottom surface (equatorial plane of the large bead). Once a firm interaction was achieved, a probe bead was trapped at higher power (480 mW before the objective) and focused at the same height.

The trap was calibrated using a combined drag-force-power spectral analysis method as described in ref [48]. This method employed a sinusoidal displacement of 150 nm at a frequency of 32 Hz. The displacement was controlled by the analog voltage outputs of a 16-bit acquisition card (PCI-6361, National Instrument) to the piezocontrollers (TPZ001, Thorlabs). The QPD recorded the voltage signals via the analog inputs of the DAQ-card at *f*_sample_ = 65536 Hz at a sampling rate for *t =* 1/8 s. The power spectrum density was averaged over 10 measurements and fitted using a custom-MATLAB least-squares fitting routine implementing a Levenberg-Marquardt algorithm. The acquisition and stiffness determination took less than 2 s. The trap stiffness was typically in the range 140–180 pN.μm^-1^ and 20–40 pN.μm^-1^ along the *x*-, *y*- and *z*- axes, respectively.

**Fig 2:**
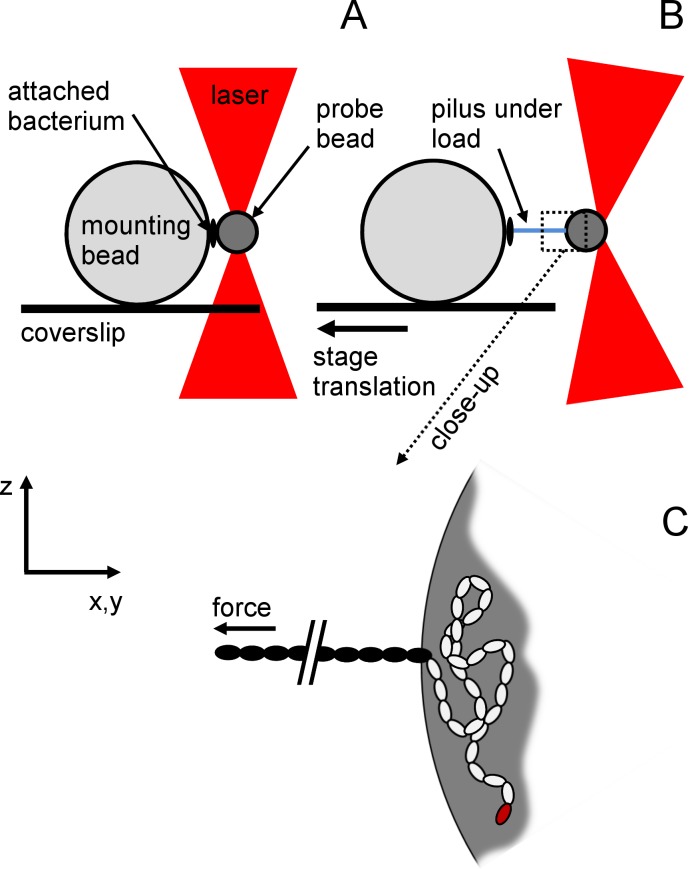
Experimental configuration of single-molecule force spectroscopy assays. Scheme illustrating the measurement configuration that was used during the experiments with no force applied (A) and under force (B) when the stage was moving. C is a close-up of B.). The pilus attached to the trapped bead is disproportionally long for reasons of depiction. The 10.5-μm mounting bead (MB) was immobilized on the coverslip while the 1-μm probe bead (PB) was trapped by optical tweezers (OT). A piliated bacterium was non-specifically attached to the MB and a pilus to the PB. When the coverslip was moved, and the trap kept in a fixed position, a force was directly exerted on the pilus. (C) Assuming adhesion to be non-specific, the most likely situation is that a portion of the pilus was attached (white subunits) and not solely the adhesion pilin (red subunit). Only a part of the pilus (black subunits) was thus subjected to the applied force.

Once the trap was calibrated, the probe bead was brought into the vicinity of the bacteria (~100 nm) for approximately 1 min (Fig 2A).

A first attempt to bring away the bead was manually made using the joystick. If the bead detached, the event was reported as “failure”, and if not, as a “success”. The total number of attempts is listed in Table 2. When the probe bead remained bound, the custom-LabVIEW program set the retraction to a translation speed of 100 nm.s^-1^. During retraction, the position of the bead was converted into force in pN using the calibrated stiffness and gave rise to a force-extension curve [49,50] as shown in Fig 2B and 2C. The sampling rate was set at *f*_sample_ = 2 kHz. The axial force (along the *z*-axis) was also recorded to control cross-talk between lateral and axial axes (see S1 Appendix and S2 Fig) and to ensure the appropriate position of the probe bead in its vertical position with the bacterium. Sometimes, the force elongation curve rapidly increased in less than a 50-nm separation distance until the bead escaped the harmonic potential of the optical trap. This event was reported as “strong attachment” in Table 2. Finally, a full force-elongation curve was obtained during this run (reported in Table 2), giving rise to a success rate *i*.*e*. the ratio of the number of elongation traces to the total number of attempts, and complemented with the strong attachment rate, *i*.*e*. the ratio of the number of attempts that required pulling forces out of the range of the set-up (typically > 200 pN) to the total number of attempts.

**Table 2:** Summary of FMOT dataset on the tested strains for which an elongation process was observed (i.e. Pil, Pil p*srtA*, Pil^ΔA^, and Pil^ΔAΔC^).

Conditions/Strain	Pil	Pil p*srtA*	Pil^ΔA^	Pil^ΔAΔC^
***Number of successful elongation data*** *(success rate (%) / strong attachment rate (%))*				
*PBS*	253 (94.4/35.6)	0 (0/100)	277 (91.1/29.8)	not tested
*+BSA 1*.*5 μM*	246 (56.1/9.5)	71 (13.1/86.8)	198 (68.9/12.3)	244 (81.8/21.9)
*+BSA 15 μM*	52 (20.3/0)	79 (26.9/74.5)	41 (26.2/0)	not tested
*+BSA 30 μM*	0 (0/0)	13 (35.5/12.3)	0 (0/0)	not tested
*L*_*p*_ values (nm)				
*PBS*	0.653 ± 0.086	failed	0.632 ± 0.082	
	0.965 ± 0.065	failed	0.967 ± 0.083	
	1.357 ± 0.085	failed	1.361 ± 0.079	
	2.748 ± 0.106	failed	2.780 ± 0.089	
*+BSA 1*.*5 μM*	0.680 ± 0.046	0.763 ± 0.187	0.739 ± 0.097	0.585*
	0.949 ± 0.082	failed	1.021 ± 0.042	0.845*
	1.406 ± 0.092	1.263 ± 0.073	1.411 ± 0.090	1.352 ± 0.116
	2.730 ± 0.114	2.795 ± 0.026	2.738 ± 0.101	2.758 ± 0.087
*+BSA 15 μM*	0.366 ± 0.019	0.724 ± 0.186		
	0.933 ± 0.119	1.037 ± 0.092	0.951 ± 0.053	
	1.360 ± 0.202	1.314 ± 0.121	1.295 ± 0.062	
	2.747 ± 0.101	2.787 ± 0.079	2.712 ± 0.097	
*+BSA 30 μM*	failed		failed	
	failed		failed	
	failed	1.422 ± 0.035	failed	
	failed	2.795 ± 0.067	failed	

A first set concerns the number of successful elongation data for the entire campaign of experiments. A second set provides the value of persistence length *L*_*p*_ extracted from fitting Eq [1] to elongation curves under different conditions of medium (PBS and PBS+1.5–30 μM BSA). When elongation curves cannot be obtained (strong attachment or no interaction), the attempt was reported as “failed”. The distribution of *L*_*p*_ exhibited four distinct modalities. Each peak was fitted with either Gaussian function or deconvoluted using multipeak convergence and reported in this table.

(*) indicates failure in multipeak fitting convergence. Values are only the position (in nm) of the highest peak.

### Modeling a sortase-assembled pilus with the Worm-Like Chain model

Lactococcal pili are hypothesized to share a common architecture with other sortase-assembled pili [8] as depicted in Fig 1. A common model for describing the force–extension response of linear polymers that undergoes thermal fluctuations is the Worm-Like Chain (WLC) model [51,52]. This model describes the polymer as a continuous flexible chain of length *L*_*c*_ [nm] with a bending stiffness, which is often expressed in terms of a persistence length, *L*_*p*_ [nm].

As there is no analytical solution to the WLC model for the entire range of forces, approximate solutions have been developed [53]. The most common approximation is the interpolated WLC, derived by Bustamante *et al*.[51], which reads:

$$F(L)=\frac{k_{B}T}{L_{p}}\left(\frac{1}{4}{\left(1-\frac{L-\delta L}{L_{c}}\right)}^{-2}-\frac{1}{4}+\frac{L-\delta L}{L_{c}}\right)-\delta F_{0},$$ [1]

in which *k*_*B*_ is the Boltzmann’s constant and *T* the absolute temperature (*k*_*B*_*T* in J or pN.nm), *L* (nm) represents the end-to-end distance of the segment of the pilus being stretched between the two bead surfaces in the direction of the elongation, δ*L* (nm) and δ*F*_*0*_ (pN) are lateral and force offsets, respectively. Lateral offset was typically in the range 0–50 nm. The force offset was obtained when rupture occurred *i*.*e*. probe bead was detached. This value was then updated with axial force response (using axial position and stiffness values) in the case of cross-talk [54] yielding a possible tilt of the pilus during extension (see S1 Fig for more details). Determination of the two offset values provided remarkable accuracy of *L*_*p*_ and *L*_*c*_ values. This analytical solution was thus used in the present work to analyze the force–extension behavior of pili produced by *L*. *lactis*.

The experimental data were then fitted with the model of Eq [1] using a custom-MATLAB least-squares fitting routine yielding a regression coefficient of *R*^*2*^ > 97%.

## Results and Discussion

### Pilus of *L*. *lactis* is highly flexible but inextensible

**Fig 3:**
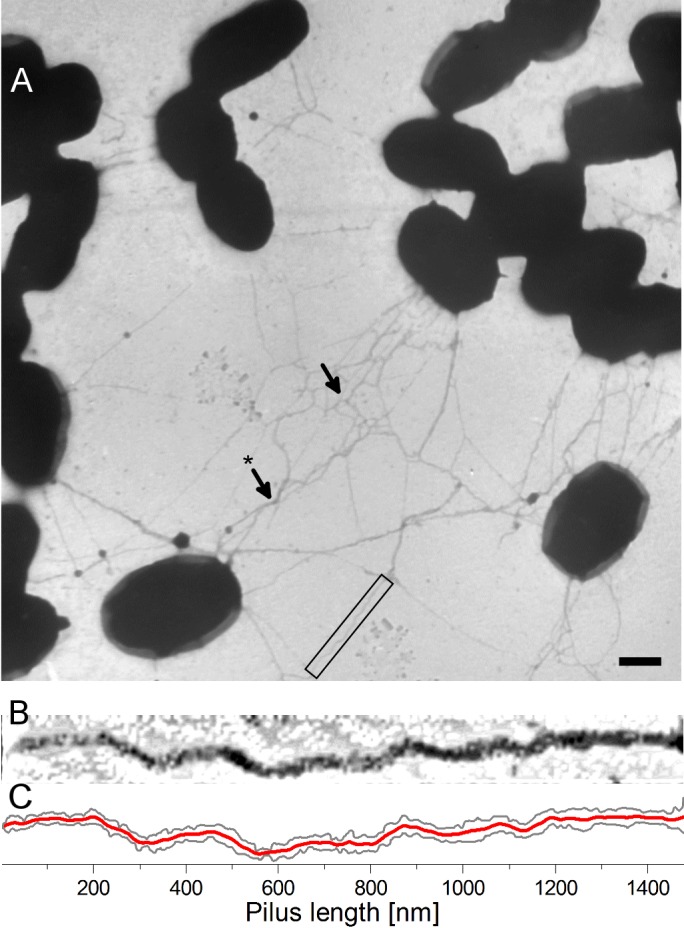
Transmission electron micrograph of Pil strain. (A) Pil strain bacterium (scale bar is 500 nm). A close-up of a pilus (black square) is shown in (B) with higher contrast and (C) image analysis of the contour.

The *pil* operon drives pilus biogenesis in *L*. *lactis* resulting in protruding filamentous structures on the bacterial cell surface that tend to tangle and wrap around each other [34]. Fibers reach up to 3 μm in length with a diameter of 5-nm as observed by atomic force microscopy. The wild-type strain have no pili on the surface. The mechanical flexibility of pili can be assessed by their persistence length. The persistence length *L*_*p*_ used in the WLC model opposes the thermal fluctuations, which tend to randomize the orientation of a polymer, to the energetic cost of bending. Alternatively, it characterizes the correlations of the tangent vectors at different positions along the polymer [55]. For a pilus, the tangent vectors of two segments are totally uncorrelated, showing that the entropic elements of free energy dominate over the bending stiffness. Fig 3A is a transmission electron micrograph showing the strain Pil–over-expressing the entire *pil* operon–which produces long appendages, forming bundles (arrow with a star), sometimes highly tangled (arrow).

The pili are very long– 1 to 3 μm length–i.e. longer than the bacterium itself. The pilus shown in Fig 3B (which is a close-up of the black rectangle) is not entirely straight, in contrast to the predicted architecture shown in Fig 1. Fig 3C shows image processing of the pilus revealing the edges (solid dark grey lines) and the mean value (solid red line). It shows that pili display high curvature that reflect a relative small persistence length. Several studies have estimated the persistence length through image observations, e.g. in *Corynebacterium diphteriae* [56] by measuring the bending angles over 50-nm segments using AFM, obtaining a persistence length of ~280 nm. The authors claimed that this method could have limitations resulting from the drying process although they assumed that the pili thermally equilibrate in the substrate plane before drying. We measured the bending angle of straight pili by analyzing the mean curve (Fig 3C—solid red line) using the first derivative to obtain its inverse tangent. In thermodynamic equilibrium, the probability *p* that the segment *ΔL* is bent by a specific angle *θ* is given by Boltzmann’s law *p*(*θ*) ∝ exp(−*U*(*θ*)/*k*_B_*T*) = exp((−*L*_p_/Δ*L*)*θ*^2^) with *U*(*θ*) the energy required to bend the segment. Consequently, the standard deviation $\sigma_{\theta }^{2}$ of the bending angles is given by $\sigma_{\theta }^{2}=\left\langle \theta^{2}\right\rangle =\Delta L/L_{p}$ We obtained an estimation of the persistence length for the Pil strain using the TEM micrographs of 36 ± 15 nm, with *σ* = 1.17 rad, over the 50-nm segments analyzed (N = 31) and 30 pili. This persistence length is relatively small compared to other types of pili, such as that obtained for *C*. *diphteriae* using the same method of analysis of the bending angles. This could result from the lower resolution of TEM images relative to those of AFM images.

**Fig 4:**
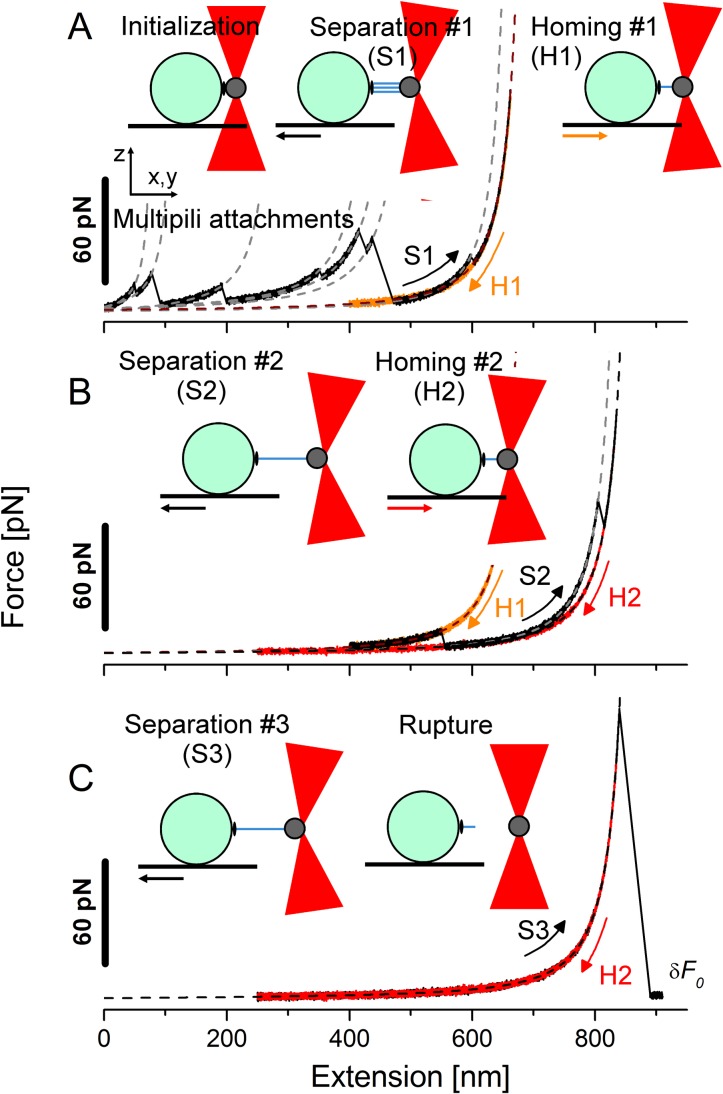
Force spectra from extension/retraction cycles experiments. Typical force spectra experiment at constant velocity on the Pil strain under conditions of 1.5 μM BSA. The steps were organized as follows: (A) the initialization, separation and homing steps giving the extension (S1) and retraction (H1) traces; (B) second separation/homing cycle with S2 and H2 and (C) third separation until rupture with S3 where the force finally dropped down to the zero force offset (δ*F*_0_).

The persistence length determines the bending stiffness of the pilus and is therefore a key parameter of its nanomechanical properties. We applied optical-tweezers force spectroscopy to more accurately determine the persistence length of a pilus attached to living bacteria–for single-pili—and to circumvent artifacts of the sample preparation such as the drying process. The Pil strain produced a substantial number of long pili (Fig 3A) and it was difficult to avoid multiple attachments. Back-and-forth cycles were performed until a single attached pilus remained (Fig 4).

A concentration of 1.5-μM BSA was typically used. Force-versus-extension spectra show a saw-tooth pattern related to consecutive unbinding during separation (Fig 4A). After an initialization step to create the interaction between the pili and the probe bead, the *Separation* step #1 gave the extension force response shown by trace S1. This trace resulted from several rupture events originating from numerous pili-bead interactions referred to as *multipili attachments* that were also monitored by the axial position of the bead (see S1 Appendix and S2 Fig for supplementary details). Each instance (solid black lines) was fitted with the WLC model given by Eq [1] (grey dashed lines). The persistence length of each fit is shown in Table 2. The retraction run (H1) retraced the previous extension curve S1 and stopped earlier (at ~400 nm). The second extension cycle with extension curve S2 (Fig 4B) resulted in another rupture. The last curve was retraced during the second retraction run (H2). The third cycle (Fig 4C) displayed no hysteresis with extension run (S3) that retraced the previous rectraction (H2), until the force dropped down to the offset value δ*F*_*0*_ after detachment of the pilus.

The acquired force increased monotonically (Fig 4A) in a non–linear manner until the force stalled at the end of each part. There was no visible plateau of constant force, in contrast to *L*. *rhamnosus* GG, possibly because *L*. *lactis* pili do not have ancillary pilin along the shaft, which gives *L*. *rhamnosus* GG pili the ability to adhere via several attachment sites [18,19]. In contrast, the curve exhibited a sharp increase in the force values due to the entropy that hampers further extension of the pilus. During the force-extension measurements, the pilus was not stretched as an individual monomer but was instead extended as a united linearized structure [57]. The WLC model that treats the pili as linear structures seems appropriate, with *R*^2^>97%. Fig 4C showed no hysteresis for long extension (up to 900 nm) nor during retraction of the pilus, indicating that no conformational changes occurred during the extension procedure. This result is consistent with the findings of Alegre-Cebollada and co-workers, showing that the isopeptide bonds of the major pilin Spy0128 produced by *S*. *pyogenes* are inextensible and thus block the unfolding of the pilin monomer [57]. The extension/retraction process was reversible as we observed no unfolding of subunits under 200 pN using FMOT. This suggests that the shortest pili that unbound operated in a “sacrificial-bond system” to leave the longest pili operational. The single-pilus force-extension curve (trace S3 in Fig 4C) was thus fitted with the WLC model and yielded a value of 2.7 nm. This value is much smaller than that obtained using TEM (mean value of 36 nm) raising the question of whether conditions of pili immobilization on a surface before drying for TEM observations are appropriate for such measurements. This value is also smaller than the predicted length of PilB (55 kDa [34] i.e. length of ~ 10 nm based on RrgB pilin from *S*.*pneumoniae*, 12-nm long [16] and Spy0128 from *S*. *pyogenes*, 10-nm long [58,59]), reflecting high flexibility of the macromolecule. The elastic cost to bend the pili under these experimental conditions was particularly low compared to the thermal energy. Therefore, such low persistence length value may be rationalized by describing the stretching of extended polypeptide segments (generated as a result of the applied force) as reported in other works [57,60] and not on the stretching of a polymer of globular units. Indeed, Alegre-Cebollada *et al*. [57] have determined *L*_*p*_ values between 0.2 and 2.3 nm to fit force spectra of Spy0128. The *L*. *lactis* pili extension data are in agreement with these findings in *S*. *pyogenes* [57] using AFM and *S*. *pneumoniae* [61] using FMOT (2.1 nm). The same order of magnitude was also observed for *E*. *coli* pili displaying a helix-like shape able to unwind under constant force using FMOT [62] (0.8 nm) or by AFM [60] (1.2–1.6 nm).

In conclusion, optical-tweezers force spectroscopy determined an *L*_*p*_ value that is close to sortase-assembling pili and other types of pili such as helixlike *E*.*coli*. FMOT gave an accurate determination of a single-pilus persistence length of 2.730 ± 0.114nm. The other early rupture events were also analyzed and their interpretation is proposed in S2 Appendix and S3 Fig. This consisted of deriving the WLC model into several WLC-behaving entities with an emphasis on persistence length. The main objective of this analysis is to interpret the force-extension spectra and differentiate whether the pilus was subjected to slip from the probe bead or unbinding (multiple attachments), giving rise to a constant *L*_*p*_ value or its increase after each rupture event, respectively.

### Nanomechanics of the pilus backbone: Sortase C and PilB are required

The Pil^ΔB^ and Pil^strC*^ strains, which lack the backbone pilin PilB and functional sortase C, respectively, have been reported to be unable to produce functional pili [34]. Consistent with these findings, force spectroscopy experiments did not show an elongation process for Pil^ΔB^ or Pil^StrC*^, behaving similarly to the negative control. Our data, together with molecular biology studies [34], confirm that sortase C is essential for the polymerization of PilB pilin subunits in *L*. *lactis*, as shown in other sortase-assembled pili of Gram-positive species such as *S*. *pneumoniae* [16,63], *S*. *pyogenes* [64], *E*. *faecalis* [65] and *Streptococcus agalactiae* [66]. Indeed, PilB is essential for the structure of the pilus backbone; no other pilin can fulfill this function. The force-elongation curves that will be discussed below are thus restricted to the strains that produce structures containing oligomerized PilB moieties, *i*.*e*. Pil, Pil p*srtA*, Pil^ΔA^ and Pil^ΔAΔC^.

**Fig 5:**
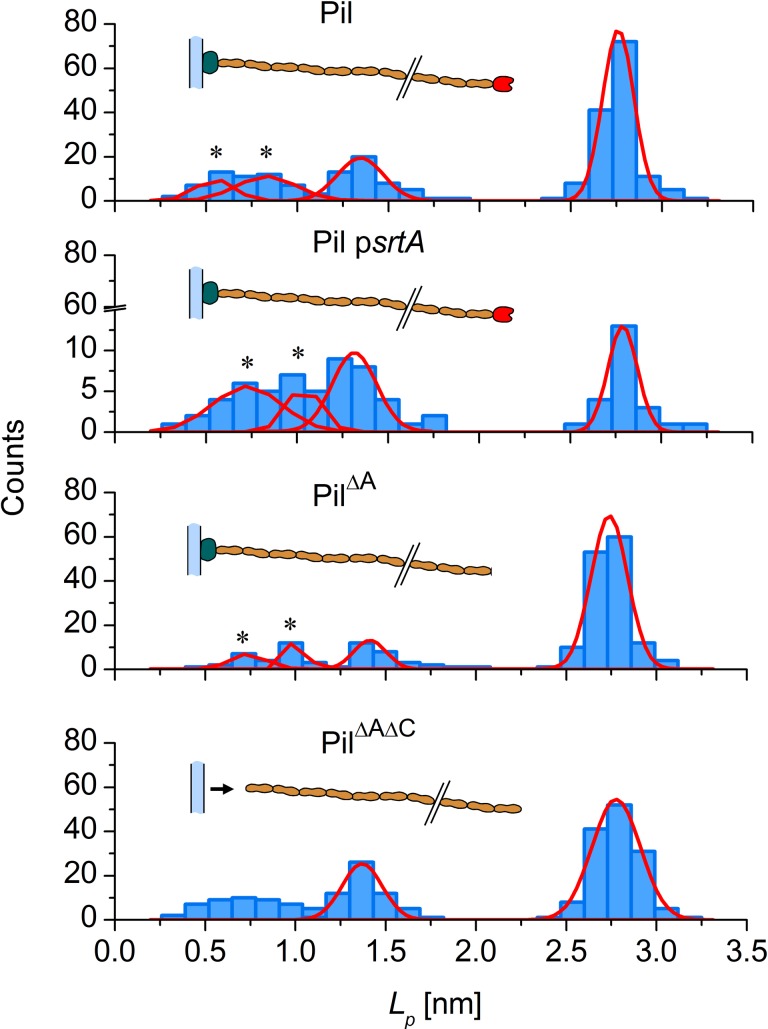
Histograms of persistence length *L*_*p*_ (nm). The positions are provided by fitting WLC model to force spectroscopy data on the Pil strain and its pili-displaying derivatives with 1.5 μM BSA or 15 μM BSA for the Pil p*srtA* strain. Data were fitted using the Gaussian function (solid line) and Gaussian multi-peak analysis (solid line and black star (*)).

Unless stated otherwise, the persistence length of the four pili-producing strains used in this study are plotted in Fig 5 by fitting Eq [1] to the full-length force-extension curves.

A concentration of 1.5-μM BSA was used for experiments with the Pil, Pil^ΔA^ and Pil^ΔAΔC^ strains, whereas 15 μM BSA was used for Pil p*srtA*. We fitted the distributions around the apparent peaks (either using Gaussian fitting or multipeak Gaussian analysis, as indicated by a black star) and revealed four values that were shared between the strains, *i*.*e*. ~2.7 nm, ~1.3 nm, ~0.9 nm and ~0.6 nm (Table 2). We deduced the value of 2.7 nm by fitting the last curve resulting from the optimized cycling procedure. This value was shared between the four strains that produced polymerized PilB under the different medium conditions (PBS and BSA).

Strains Pil^ΔA^ (that does not produce PilA tip pilin) and Pil^ΔAΔC^ (that does not produce PilA tip pilin or PilC anchoring pilin) both produce pili. Only Pil^ΔA^ pili gave results for the extension process (Table 2, Fig 5), whereas Pil^ΔAΔC^ did not. This was expected because the absence of the anchoring PilC pilin in this strain results in the release of PilB polymers into the extracellular medium, demonstrating that PilC is required for the attachment of pili to the cell wall. We took advantage of this feature to perform elongation attempts on bacteria-free pili. The strain Pil^ΔAΔC^ was prepared as explained in *Materials and Methods*. Pili released into the culture supernatant were recovered using previously reported protocols for P-pili producing *E*. *coli* [37,38]. The amount of BSA was optimized to avoid aggregation (1.5 μM) and attachment to the mounting and probe beads was achieved. We obtained a sufficient amount of elongation data to characterize the free pili (n = 244). The absence of the PilA tip pilin did not appear to substantially affect the biomechanical properties of the strain Pil^ΔA^ or pili from Pil^ΔAΔC^. The value of 2.7 nm was conserved among the strains indicating that PilA, which is the first secreted and assembled pilin in the pilus biogenesis process, does not play a role in the ultrastructure and nanomechanics of the final pilus. This result reinforces our above conclusions on the key role of the PilB pilin in *L*. *lactis* pili nanomechanics.

The Pil p*strA* strain, which overexpresses pili with a certain amount released into the surrounding medium [34], was examined further. We never succeeded to produce elongation data under PBS conditions with this strain because the probe bead stuck to the bacterium. The behavior of this strain in the presence of BSA was different from that observed for other strains (Table 2). BSA is commonly described to suppress non-specific binding in favor of specific receptor-ligand interactions (e.g.[67]) by mainly neutralizing hydrophobic interactions [68]. The percentage of success was low for strain Pil p*strA*, regardless of the BSA concentration: for example, 13.1% in 1.5 μM BSA compared to 56.1% (Pil) with only 2–3 single-pilus attachments (Table 2). The force needed to overcome this strong attachment was probably too large for FMOT (probably in the range of nanoNewtons). The number of measurements was still small when the concentration of BSA was increased to 30 μM (n = 13, Table 2). The intermediate BSA concentration of 15 μM resulted in profiles similar to those achieved with the Pil strain. Altogether, our results show that the number of measurements of the last peak value (~2.7 nm) increased with the amount of BSA which correlated with the number of pili involved in the force spectra. The use of BSA tended to minimize the amount of strong attachment events and favored the single pilus configuration. In a previous study, Klinth *et al*. [69] used BSA (from 2.8 to 11.3 μM) to measure the influence of the PapD chaperone protein on refolding of P pili produced by *E*. *coli*. BSA, even at 11.3 μM, did not affect the refolding process of the helix-like pilus when subjected to external force, where only few disturbances were detected. Our results also suggest that BSA did not impede the linearization process and did not thus influence the persistence length.

Our findings with the Pil p*strA* strain clearly show that the use of p*strA* on the strain lacking expression of class A sortase not only reactivated the function of the sortase but also resulted in the secretion of more pili. The pili produced had identical biomechanical properties to those of the Pil strain. The presence of BSA reduced the number of attachment events between pili suggesting that BSA either a contributes to site-specific adhesion and/or homophilic adhesion. The second possibility was previously shown by studying the biofilm-forming properties of the Pil p*strA* strain [34]. The biofilms were highly reticulated, heterogeneous, rough, and aerial [34] due to the high density of pili and their subsequent steric interactions that somehow maintained foamy aggregates.

### Nanomechanics of *L*. *lactis* pili are a key player in its interactions with the environment: comparison with other types of pili

*L*. *lactis* pili can only extend ~ 1 time their original length (Fig 4), whereas the pili expressed by uropathogenic *E*. *coli* strains can extend more than 5 times their length [50] under FMOT force. The *L*. *lactis* sortase-assembled pilus thus behaves as a barely extensible biopolymer. This may be attributable to an evolutionary artifact or originate from the different shear forces that prevail in the diverse sites of bacterial colonization. This has been proposed by Mu and co-workers who observed that Hib pili on *Haemophilus influenzae* could not overextend, suggesting that regions of intense shear forces, such as recurrent coughs and sneezes, favor the persistence of bacteria displaying inextensible pili [70]. However, recent work [71] compared the biomechanical properties of pili produced by uropathogenic (UPEC) and enterotoxigenic (ETEC) *E*. *coli*. The pili share common quaternary structure and the ability to unwind under constant force. Their ecological niches reflect their property to unwind because they are subjected to different types of forces. For example, UPEC are found in the ureter and need to withstand periodic shear forces due to urine boluses. The rewinding kinetics allowing the pili of UPEC to act as reversible structures have been shown to be rapid [72,73]. CS20 pili on ETEC [74] can be unwound to up to eight times their initial length by a low unwinding force. Peristaltic motion in the ileum generates smooth back-and-forth shear forces [75], suggesting that evolution has resulted in pathogenic bacteria with dedicated shock absorbers. In contrast, *L*. *lactis* belongs to Gram-positive bacteria with sortase-assembled subunits. Here, we have shown that such pili are not wound as those found in UPEC/ETEC strains and in turn cannot respond to shear force solely by unwinding. A possible adaptive strategy would be to form a biofilm community, notably through pili-mediated homophilic adhesion which can help in building a thick fortress. Indeed, the pili-displaying strain Pil has been shown to be able to form an aerial and reticulated biofilm, solely due to the presence of pili with respect to its negative control [34]. In addition, pili, as observed in Fig 3A, tend to form bundles or a mesh-like structure. This suggests a high affinity between pili. Indeed, *L*. *rhamnosus* GG pili exhibit ancillary proteins along the backbone that improve homophilic adhesion and display a force plateau when exposed to an external force [18]. The unzipping mechanism can easily be compared to that present in UPEC/ETEC strains that break their layer-to-layer bonds and thereby dissipate energy. This comparison has been presented in a previous publication on T4 pili of *S*. *pneumoniae* [61] which share a similar architecture with that produced by *L*. *lactis*.

## Conclusion

We analyzed the persistence length of pili produced by *L*. *lactis* reflecting their morphological and biomechanical properties. We have proposed a technical and analytical methodology to provide a reliable interpretation of certain aspects of their structural/nanomechanical relationship. This methodology consisted of using Force-Measuring Optical Tweezers to apply minute forces on several pili until some detached leaving a single pilus, followed by the exploitation of an experimental procedure, i.e. directly at the single-pilus scale using an interaction-screening protein (bovine serum albumin). Our methodology allowed us to investigate the functionality of the different pilins needed to form a pilus. We have clearly established the combined role exerted by sortase C and the backbone pilin PilB. In contrast, the tip pilin PilA was shown to be nonessential for pilus nanomechanics.

In future work, these mechanistic insights into the nanomechanics *L*. *lactis* pili will be strengthened by investigating the adhesion properties of piliated *L*. *lactis* under shear flow on different surfaces such as mucins, to propose further applications of piliated lactococci for functional food, mucosal vaccines, or therapeutic drug delivery.

## Supporting Information

S1 Appendix Axial monitoring of beads during force spectroscopy experiments. (DOCX)

S2 Appendix Proposal of derivations of the WLC model. (DOCX)

S1 Fig Defocusing effect from the linearization of the pilus during elongation. Sketch depicting extension routine of a single pilus (A). (B) At rest, the pilus is somehow folded but the two ends are not located at the same height on the mounting bead and the probe bead. (C) When the stage starts to move, the pilus linearizes and the force response is monitored, revealing the bending stiffness. (D) Once the pilus is fully linearized, the forces equilibrate and tend to defocus the bead in order to align the pilus in the same plane. This effect was monitored along the z- axis.
(TIF)

S2 Fig Force-versus-elongation curve and axial monitoring. (A) Force-versus-elongation curve (solid line) fitted with the WLC model (dashed gray line). The probe bead was set free, the force dropped down to a zero offset δ*F*_*0*_. (B) Axial position of the probe bead during the extension process. When the force reached the zero offset, the equilibrium position of the bead was indicated by δ*z*_0._
(TIF)

S3 Fig Scenarios describing force-versus-elongation spectra displaying multiple rupture events. Example of force spectra on the Pil strain (A, C, E) with successive values of *L*_*p*_ and *L*_*c*_ (B, F and D), respectively. During the extension process, the force rapidly increased and rupture events occurred: either a pilus partially detached and therefore gained sequentially an amount of contour length Δ*L*_*c*_ depicted in (G) or a pilus detached from the bead in case of multipili attachments (black stars*) illustrated by (H). (G) and (H) provide possible scenarios illustrating the two cases as slipping and multipili events. (G) involved a single pilus, some parts are colored to highlight the slipping effect. (H) involved three independent pili (yellow, blue and red). When a curve was fitted using the WLC model (dashed black line), the fitted curve was designated using a capital roman letter (*e*.*g*. *IV*). The extension range of the curve is represented by yellow/white shading (B, D, F) for easier reading of the plots.
(TIF)
