# Supplementary material for: The Nanomechanical Properties of Lactococcus lactis Pili Are Conditioned by the Polymerized Backbone Pilin
Source: PLoS One. 2016 Mar 24;11(3):e0152053. doi: 10.1371/journal.pone.0152053 (PMC4806873; doi:10.1371/journal.pone.0152053)
Supplement: S1 Appendix — (DOCX) [file pone.0152053.s001.docx]

**S1 APPENDIX: Axial monitoring of beads during force spectroscopy experiments**

S2A Fig shows a typical curve, obtained with the Pil strain in 1.5 µM BSA and fitted to the WLC model (gray dashed line). When the pilus detached from the probe bead, the force dropped down to the zero-force offset value *δF_0_*. The distance covered during this process is reflected by the deflection of the trap, from the position under force to the equilibrium position, typically ~ 200 nm. The axial position of the bead was monitored by the quadrant photodiode to track eventual tilting or cross-talk between axes (S2B Fig). As an example, when the probe bead was not exactly focused at the same height of the attaching site of the pilus on the cell wall, the pilus under load was not fully horizontal resulting in a tilt. S1 Fig depicts such a scenario. The pilus is anchored to the bacterium on one end and attached to the probe bead on its other end. The two attachment sites are not exactly at the same height (S1A and S1B Figs). During the stage movement, the pilus unfolded during bead separation until it became fully linearized, tending to defocus the probe bead, and reached equilibrium. The position of the bead compensated for this angle to align the pilus on the same plane (S1D Fig). The first part of the curve (S1D Fig) gave rise to a defocus of 200 nm from the initial position of the calibration. In the next segment, a pilus probably became totally or partially unbound, modifying the angle. The probe bead then returned to its initial position.
