# Supplementary material for: The Nanomechanical Properties of Lactococcus lactis Pili Are Conditioned by the Polymerized Backbone Pilin
Source: PLoS One. 2016 Mar 24;11(3):e0152053. doi: 10.1371/journal.pone.0152053 (PMC4806873; doi:10.1371/journal.pone.0152053)
Supplement: S2 Appendix — (DOCX) [file pone.0152053.s002.docx]

## S2 APPENDIX: Proposal of derivations of the WLC model

The main purpose of the present work is to characterize the nanomechanical properties of *L. lactis* pili when exposed to a constant force. This is achieved by fitting the WLC model given by Eq. [1] to the experimental force-elongation curve typically represented by trace S3 in Fig 4C after the multiple attachments were eliminated. However, only the early instances have been fitted and the corresponding persistence length values are shown in Table 2 and can also be seen on the histograms in Fig 5. The persistence length values were different from the given value of 2.7 nm. This appendix proposes several putative mechanisms illustrated by experimental data.

The assumption that all pili exhibit the same persistence length $L_{p}^{\text{single}}$(nm) is reasonable because they are made of the same PilB building blocks. When several pili *i* with contour length *L_c,i_* share the external force from the probe bead, the multipili response *F^TOT^* can be described as:

$F^{TOT}(L)=\frac{k_{B}T}{L_{p}^{\text{single}}}\sum_{k=i}^{n} \left[\frac{1}{4}\left(1-\frac{L}{L_{c,i}}\right)^{-2}-\frac{1}{4}+\frac{L}{L_{c,i}}\right].$ S.1

In the case of widely dissimilar contour lengths, the summation can be approximated by the value of the bracket for the shortest pilus (whose contour length is *L_c,<_*). This implies that the expression for the multipili response could be described as:

$F^{TOT}(L)=\frac{k_{B}T}{L_{p}^{\text{single}}}\left[\frac{1}{4}\left(1-\frac{L}{L_{c,<}}\right)^{-2}-\frac{1}{4}+\frac{L}{L_{c,<}}\right].$ S.2

Assuming that pili are identical and display the same contour length and persistence length $L_{p}^{\text{single}}$ (nm), the net force described by the WLC model is distributed among the pili under load and governed by an equivalent persistence length $L_{p}^{eff}$ (nm) of the pili bundle, referred to as the effective persistence length throughout this discussion. Making use of individual WLC entities with this parameter, the measurements lead to the following net force:

$F^{TOT}=\frac{k_{B}T}{L_{p}^{eff}}\left(\frac{1}{4}\left(1-\frac{L}{L_{c}}\right)^{-2}-\frac{1}{4}+\frac{L}{L_{c}}\right),$ S.3

$\frac{1}{L_{p}^{eff}}=\sum_{1}^{n} \left(L_{p}^{(n)}\right)^{-1}=\frac{n}{L_{p}^{\text{single}}}.$ S.4

This leads to a circuit-like system with resistors arranged in parallel. The total resistance of a set of resistors in parallel is found by summing the reciprocals of the resistance values. This equation means that when a pilus unbinds, the persistence length of the system increases. Given *L_c_*, the generalized net force *F* (until a pilus detaches decreasing *n*) yields:

$F=\sum_{i}^{n} F_{i}=\frac{nk_{B}T}{L_{p}^{\text{single}}}\left(\frac{1}{4}\left(1-\frac{L}{L_{c}}\right)^{-2}+\frac{L}{L_{c}}\right),$ S.5

This differs from Eq. S2 by the factor “*n*”.

In the “real” case, the pili were not the same length. Under force spectroscopy conditions (Fig 2), *i.e.* the pilus non-specifically stuck on the probe bead, it is assumed that only a part of the shaft of the longest pili was under tension (Fig 2C). We hypothesized that all pili were under tension and Eq. S5 was tested on experimental data to see whether the assumptions obeyed Eq. S2 (case A) or Eq. S5 (case B). In case A, the persistence length would vary (increase or decrease) each time the shortest pilus became unbound (the one that dictated the contour length and persistence length). In case B, each time the shortest pilus detached, the persistence length would have increased gradually or at least remained constant. In both cases, the apparent contour length of the system should increase. We provide below an analysis of the raw data obtained on the Pil strain under the 1.5 µM BSA condition from three examples to facilitate the interpretation of the data and to obtain an accurate value for one single pilus. These examples represent experimental force-extension runs that were not subjected to back-and-forth cycles (Fig 4) for which the curves displayed many rupture instances. The curves were fitted using Eq. only by keeping track of *L_p_* and *L_c_* between each instance. The value of *L_p_* after each instance should satisfy one of the two cases.

The three typical examples of force spectra are depicted and discussed below. All cases displayed a saw-tooth pattern, and each curve was fitted using the WLC model. Each example is composed of a force spectrum (S3A, S3C, and S3E Figs) and the variation of *L_p_* and *L_c_* as a function of the extension (S3B, S3D, and S3F Figs).

The first example (S3A and S3B Figs) displayed five discontinuities (*I-V*) followed by complete detachment reducing the force down to the zero offset *δF_0_* (i.e., probe bead free floating). In S3B Fig, *L_p_* remained constant and *L_c_* increased each time a discontinuity occurred.

The second example displayed six discontinuities (*I-VI*) and a force offset. In this case, *L_p_* increased each time a discontinuity occurred, and became constant after discontinuity *IV*. *L_c_* increased gradually each time a discontinuity occurred as in the first example.

The third example exhibited eight discontinuities (*I-VIII*). In this case, both *L_p_* and *L_c_* increased each time a discontinuity occurred.

We suggest the following scenario, based on the WLC-based analysis proposed by Eq.S5, for the single-pilus versus multipili mechanisms (depicted by the schemes in S3G and S3H Figs, respectively). The bacterium was attached to the mounting bead via electrostatic bonds mediated by poly-L-lysine coating (Fig 2A). This creates an interaction with the bacterial cell that is substantially stronger than that between the probe bead and the pili, which ensures that the bacterium remains properly fixed during the experiment.

In the first case, the persistence length remained constant at a value of ~2.7 nm (S3B Fig). This value was already reported in Fig 5 and is attributed to the most probable single-pilus configuration.) We suggest that the pilus is non-specifically attached to the probe bead (S3G Fig) to explain the increase in contour length (S3B Fig). Only a portion of the shaft would thus be under load as mentioned above. At certain force levels, another portion unbinds, giving rise to a longer pilus under load. This gain is illustrated by *ΔL_c_^(I)^*. In the next curve (*II*), another portion unbinds, resulting again in a longer pilus, as shown by the gain *ΔL_c_^(II)^*. This example provides a single-pilus configuration with the accurate determination of its persistence length.

In the second case, curves *I* to *III* revealed successive *L_p_* values of 0.68, 0.95 and 1.41 nm (S3D Fig). From curves *IV* to *VI*, *L_p_* remained constant with a value of ~2.7 nm (S3D Fig), reflecting the persistence length of an individual pilus. According to Eq.S4, *L_p_* of the ensemble behaves as ~1/*n* with the number of pili *n*. S3H Fig provides a probable multipili scenario of curves *II-IV*; curves *IV, III* and *II* reflect a single-pilus configuration, two pili with half of a single-pilus persistence length and three pili with a third of a single-pilus persistence length, respectively. Curve *I* was not interpreted because the separation distances were too short. As schematically shown (S3H Fig), once the piezostage starts to laterally separate the bead from the bacterium, the shortest pilus (red) detaches at a given extension distance. The detachment of one of the three pili would not have an effect on the contour length, because no length is added, but it would have a direct impact on the value of the persistence length. There are fewer degrees of freedom for the system of a single pilus, relative to the original system with three pili, and the detachment of a pilus thus induces a decrease in the entropy and a subsequent increase in the persistence length. As the piezostage continues to move the probe bead away from the bacterium, the second pilus (blue) detaches. During a discontinuity, the force does not drop to near the normalized value of zero force. This confirms that all pili that remain attached are still under tension, given the extreme flexibility of the pili and the strong electrostatic interactions. The first two cases are representative results that demonstrate i) single-pilus extension and ii) single-pilus extension accompanied by several sacrificial pili that detach during extension.

We propose a third case (S3E and S3F Figs) to further illustrate the sensitivity of our interpretation method. Keeping track of the *L_p_* and *L_c_* values obtained from curves *I to VIII* (fitted using Eq.), revealed that only the two last curves displayed *L_p_* values of ~2.7 nm. This gradual increase demonstrates the successive detachment of pili along with a sequential increase in contour length. As an example, curves *V* and *VI* gave two constant values of *L_p_* = 1.31 ± 0.02 nm. Using Eqs.S3 and S4, this value is half that of a single-pilus based on the histograms in Fig 5. We propose that this part of the spectrum involved two pili.

These three examples show that two scenarios, *i.e.* single-pilus versus multipili attachment, may be occuring during the extension process of pili produced in *L. lactis*. Due to the presence of at least four distinct distributions of *L_p_* (Fig 5), the multipili attachment scenario appears to be more prevalent during the force spectroscopy experiments. The determination of the single pilus *L_p_* value is challenging as there is a difference between the single pilus *L_p_* value and the effective one (0.9 to 2.7 nm in some cases).
