# Supplementary figures and images for: The Nanomechanical Properties of Lactococcus lactis Pili Are Conditioned by the Polymerized Backbone Pilin
Source: PLoS One. 2016 Mar 24;11(3):e0152053. doi: 10.1371/journal.pone.0152053 (PMC4806873; doi:10.1371/journal.pone.0152053)

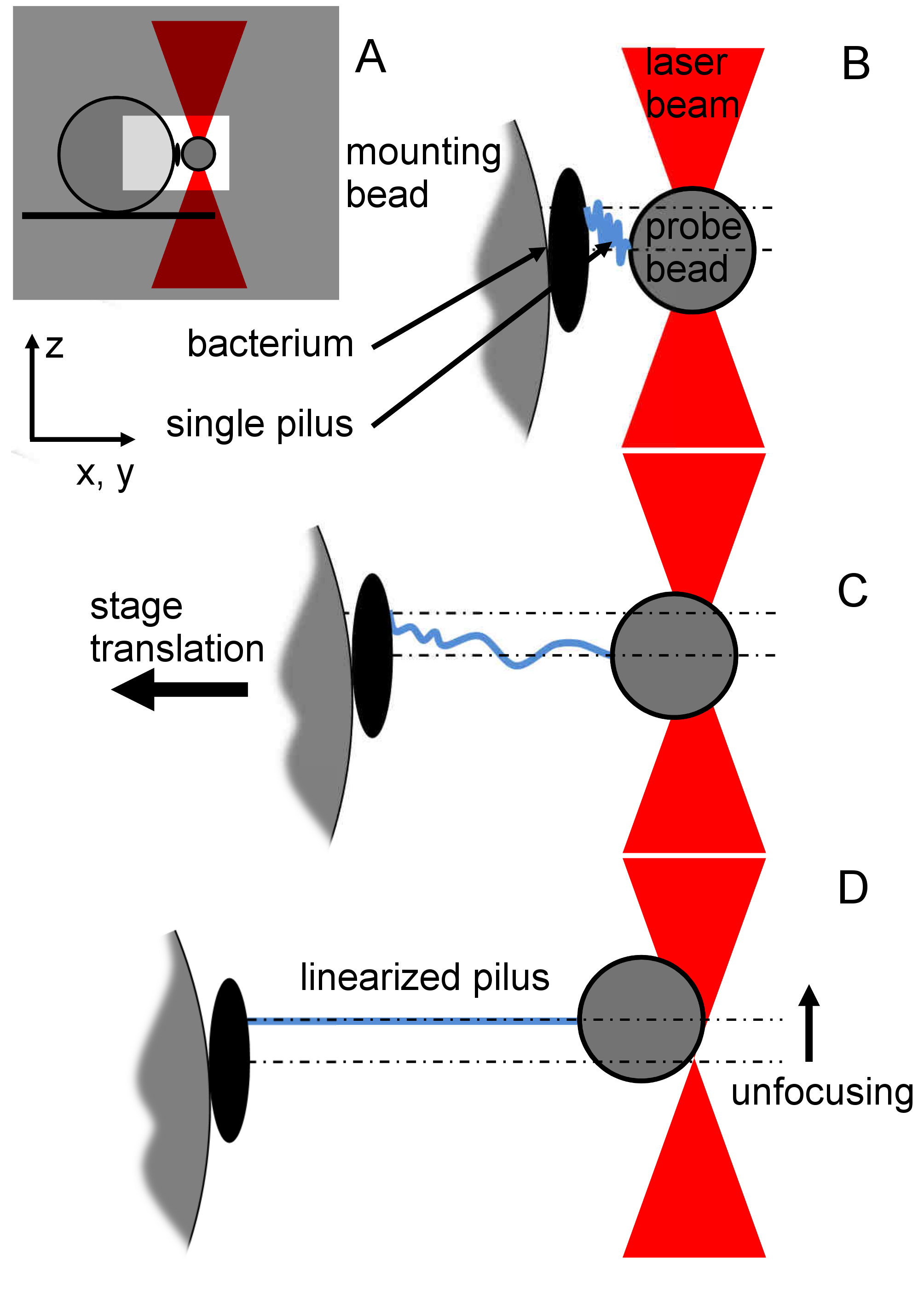

Supplement: S1 Fig — Sketch depicting extension routine of a single pilus (A). (B) At rest, the pilus is somehow folded but the two ends are not located at the same height on the mounting bead and the probe bead. (C) When the stage starts to move, the pilus linearizes and the force response is monitored, revealing the bending stiffness. (D) Once the pilus is fully linearized, the forces equilibrate and tend to defocus the bead in order to align the pilus in the same plane. This effect was monitored along the z- axis. (TIF) [file pone.0152053.s003.tif]

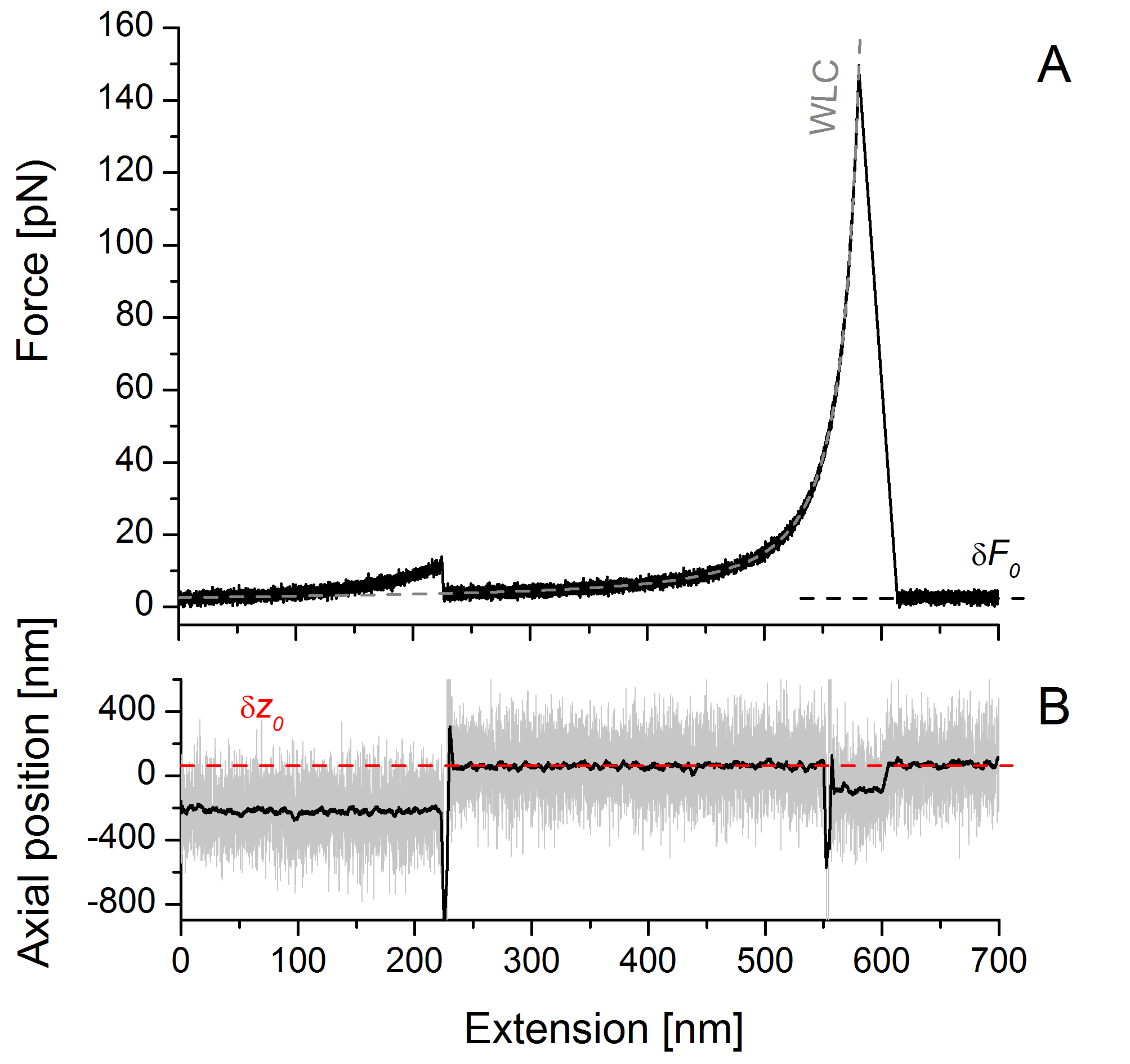

Supplement: S2 Fig — (A) Force-versus-elongation curve (solid line) fitted with the WLC model (dashed gray line). The probe bead was set free, the force dropped down to a zero offset δF0. (B) Axial position of the probe bead during the extension process. When the force reached the zero offset, the equilibrium position of the bead was indicated by δz0. (TIF) [file pone.0152053.s004.tif]

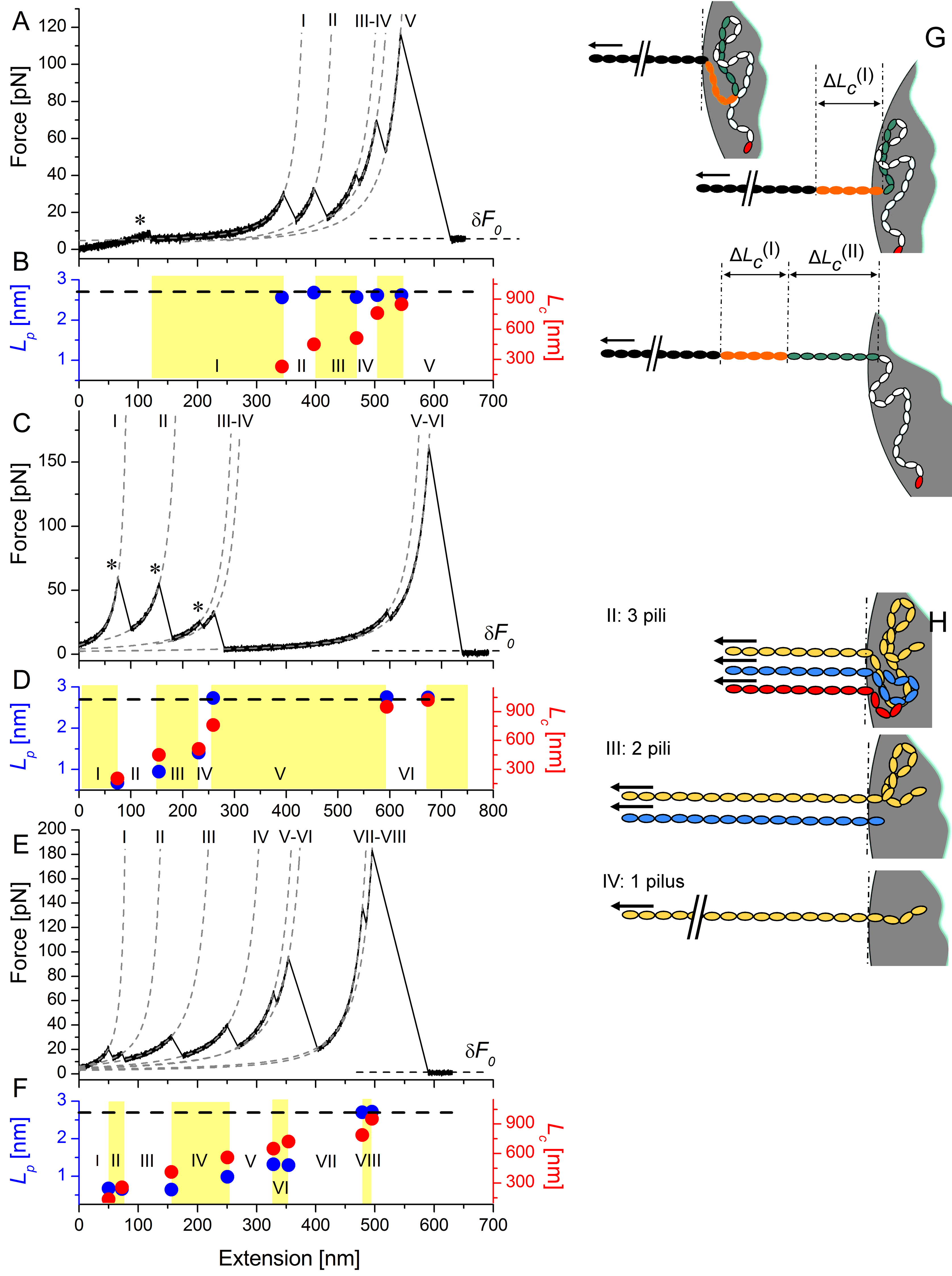

Supplement: S3 Fig — Example of force spectra on the Pil strain (A, C, E) with successive values of Lp and Lc (B, F and D), respectively. During the extension process, the force rapidly increased and rupture events occurred: either a pilus partially detached and therefore gained sequentially an amount of contour length ΔLc depicted in (G) or a pilus detached from the bead in case of multipili attachments (black stars*) illustrated by (H). (G) and (H) provide possible scenarios illustrating the two cases as slipping and multipili events. (G) involved a single pilus, some parts are colored to highlight the slipping effect. (H) involved three independent pili (yellow, blue and red). When a curve was fitted using the WLC model (dashed black line), the fitted curve was designated using a capital roman letter (e.g. IV). The extension range of the curve is represented by yellow/white shading (B, D, F) for easier reading of the plots. (TIF) [file pone.0152053.s005.tif]
